# Supporting return to work after psychiatric hospitalization—A cluster randomized study (RETURN-study)

**DOI:** 10.1192/j.eurpsy.2022.2357

**Published:** 2023-01-09

**Authors:** Johannes Hamann, Anne Lang, Lina Riedl, Daniela Blank, Monika Kohl, Adele Brucks, David Goretzko, Markus Bühner, Tamara Waldmann, Reinhold Kilian, Peter Falkai, Alkomiet Hasan, Martin E. Keck, Michael Landgrebe, Stephan Heres, Peter Brieger

**Affiliations:** 1 Department of Psychiatry and Psychotherapy, Klinikum rechts der Isar, Technische Universität München, München, Germany; 2 Bezirksklinikum Mainkofen, Deggendorf, Germany; 3 Kbo-Isar-Amper-Klinikum, Haar, Germany; 4 Psychological Methods and Assessment, Ludwig-Maximilians-Universität München, München, Germany; 5 Klinik für Psychiatrie II am BKH Günzburg, Sektion Gesundheitsökonomie und Versorgungsforschung, Günzburg, Germany; 6 Department of Psychiatry and Psychotherapy, Klinikum der Ludwig-Maximilians-Universität München, München, Germany; 7 Medical Faculty, Department of Psychiatry, Psychotherapy and Psychosomatics, Bezirkskrankenhaus Augsburg, University of Augsburg, Augsburg, Germany; 8 Department of Psychiatry, Psychotherapy and Psychosomatics, Klinik Seewis, Seewis, Switzerland; 9 Department of Psychiatry and Psychotherapy, kbo Lech-Mangfall-Hospital, Agatharied, Germany

**Keywords:** Mental health, rehabilitation, return to work

## Abstract

**Background:**

If people with episodic mental-health conditions lose their job due to an episode of their mental illness, they often experience personal negative consequences. Therefore, reintegration after sick leave is critical to avoid unfavorable courses of disease, longer inability to work, long payment of sickness benefits, and unemployment. Existing return-to-work (RTW) programs have mainly focused on “common mental disorders” and often used very elaborate and costly interventions without yielding convincing effects. It was the aim of the RETURN study to evaluate an easy-to-implement RTW intervention specifically addressing persons with mental illnesses being so severe that they require inpatient treatment.

**Methods:**

The RETURN study was a multi-center, cluster-randomized controlled trial in acute psychiatric wards addressing inpatients suffering from a psychiatric disorder. In intervention wards, case managers (RTW experts) were introduced who supported patients in their RTW process, while in control wards treatment, as usual, was continued.

**Results:**

A total of 268 patients were recruited for the trial. Patients in the intervention group had more often returned to their workplace at 6 and 12 months, which was also mirrored in more days at work. These group differences were statistically significant at 6 months. However, for the main outcome (days at work at 12 months), differences were no longer statistically significant (*p* = 0.14). Intervention patients returned to their workplace earlier than patients in the control group (*p* = 0.040).

**Conclusions:**

The RETURN intervention has shown the potential of case-management interventions when addressing RTW. Further analyses, especially the qualitative ones, may help to better understand limitations and potential areas for improvement.

## Introduction

Mental illnesses are among the most common diseases worldwide [1, 2]. In Germany, 28% of the population aged 18–79 has suffered from a mental illness within the last 12 months [3]. Consequently, cases of inability to work caused by mental illness are frequent and the related economic burden is immense [4].

Mental illness and employment interact in many ways. On the one hand, mental illnesses prevent persons from finding and keeping regular employment [5] and are responsible for economic consequences. On the other hand, work and employment have positive effects on the course of mental illnesses [6, 7]. In fact, it has been argued that “work is a critical mental health intervention” [8]. Accordingly, obtaining and maintaining jobs for people with mental illness is challenging, but can be an important prerequisite for a good prognosis.

People with episodic mental health conditions and fixed employment are a large group of society, however, they are often neglected in clinical practice and research [9]. If those persons lose their job due to an episode of their mental illness, they do not only experience personal negative consequences (e.g., missing day structure and loss of income), but also cause a serious societal problem due to resulting follow-up costs (e.g., sickness benefits, unemployment benefits, reduced societal contributions, etc.). A particularly vulnerable time to lose a job is that of an acute mental health crisis, which may require hospital inpatient treatment [10]. Hospital treatment and longer periods of sick leave interrupt job routines as well as interpersonal relationships at the workplace, making reintegration difficult. A successful reintegration of those patients after the acute episode of illness would therefore be an important prognostic factor for the further course of the disease. A failed reintegration in contrast would be a risk factor for a less favorable course of the disease, longer inability to work, long payment of sickness benefits, and unemployment.

Existing return-to-work (RTW) programs have nearly exclusively focused on “common mental disorders” (e.g., stress-related disorders, anxiety disorders, and minor depression). Their approaches vary substantially including, for example, cognitive behavioral therapy (CBT) programs [11], rehabilitation programs [12], case management [13], or, quite frequently, complex interventions combining clinical and work-directed measures [14]. Most interventions were very elaborate and costly, however, not yielding completely convincing effects regarding RTW [2]. In addition, existing studies often systematically excluded those persons with more severe mental illnesses such as schizophrenia, bipolar disorder, borderline personality disorder, or major depression [2]. It was therefore the aim of the RETURN study to evaluate an easy-to-implement return to work intervention specifically addressing persons with mental illnesses being so severe that they require inpatient treatment.

## Methods

### Study design

The study was designed as a multi-center, cluster-randomized controlled trial in acute psychiatric wards in southern Germany addressing inpatients suffering from a psychiatric disorder [15]. In intervention wards case managers (RTW experts) were introduced who supported patients in their RTW process, while in control wards treatment as usual (TAU) was continued. The scientific rationale was that the introduction of RTW experts puts more focus on the workplace-related needs of patients, leading to a better usage of existing resources as part of a work-related discharge management, and thus to a more successful return to the workplace.

### Setting and participants

The study was implemented on *n* = 28 acute wards (=clusters) in seven psychiatric hospitals in the greater Munich area. All patients admitted to these wards were consecutively recruited for the trial when they fulfilled the following inclusion criteria:

1. Age 18–60 years.
2. Diagnosis of a mental illness (ICD-10 Chapter F2, F3, F4, or F6, i.e., psychotic disorders, affective disorders, anxiety disorders, obsessive-compulsive disorders, or personality disorders).
3. Admission to inpatient treatment.
4. Existing employment.

Exclusion criteria were mental retardation, insufficient proficiency in German language to engage with the case manager, employment in a mini-job (salary of less than 450€), and a main diagnosis of an organic mental disorder (F0), substance abuse (F1), or an eating disorder (F5).

### Intervention and control condition

The intervention consisted of the implementation of RTW experts in the intervention wards [16]. The RTW experts supported patients and mental health care professionals in the wards in all areas related to the possible return of patients to the workplace. The RTW experts acted as case managers, who first clarified the specific needs of the clients (i.e., participating patients who might return to an existing workplace), subsequently communicated these needs to all people involved in the treatment process and then developed an RTW plan together with the client. Within the study four persons acted as RTW experts. Requested qualifications for this job were similar to those of social workers, who usually support patients in their RTW-process. Thus, we selected staff familiar with the principles of case management, counseling, and motivational interviewing. All four RTW experts (two social workers, one psychologist, and one educationist with extensive experience in human resources) were extensively trained to follow a manualized procedure [16], that included all of the required skills.

According to the manual RTW experts offered five structured sessions to patients during the inpatient stay (Session 1: Assessment; Session 2: Information about RTW measures/legal framework; Session 3: Disclosure; Session 4: Planning of RTW talks with supervisors; and Session 5: Planning of further support by RTW expert after discharge from hospital) and three sessions after discharge from hospital (including the possibility of a joint discussion with the employer). The services of the RTW experts could be claimed by the patients during inpatients stay and for up to half a year after discharge. The minimum of intervention was defined as at least two meetings between RTW experts and patients—one appointment during the inpatient stay and one after release (“per protocol”). RTW experts aimed at activating existing support services of the hospitals (e.g., work therapy, cognitive training, and socio-educational counseling) and to offer specific support (e.g., joint meetings between clients, employers, and RTW experts), if there was a limitation of resources. Overall, this intervention was oriented toward the competencies (and capacities) of social workers in mental health in order to facilitate implementation into routine care in case of positive study results.

Staff (and patients) of the control wards acted under “TAU” conditions. To avoid contamination bias as much as possible (i.e., staff or patients from control wards getting to know about the RTW intervention), wards were chosen in a way so that there was no overlap in personnel and that there was no regular patient transfer between wards.

### Data collection and outcomes

The same data were collected at the same time points in the intervention group and control group [15]. In the present analysis, we report on the primary outcome (number of days at work within 12 months after discharge according to patients’ self-report) and related secondary outcomes. These include days at work at 6 months, the number of patients returned to work at 6 and 12 months, and sick leave days at 6 and 12 months.

In addition, we obtained several potentially mediating factors such as the uptake of available support strategies (e.g., stepwise reintegration into work or specific services for people with disabilities). Furthermore, we documented the patients’ subjective readiness to return to their workplace and their feeling of being supported in this process by using self-rated visual analog scales. Finally, clinical data on illness severity (Clinical Global Impression, CGI, HONOS), social functioning (GAF), quality of life (EuroHisQol), and relapses (patient self-report) are reported.

### Randomization and blinding

Randomization was done at cluster level (*n* = 14 wards each to the intervention group or control group) to minimize contamination effects [17]. Therefore, pairs of comparable wards (number of patients, distribution of diagnoses, staff, etc.) were determined by the principal investigators and then one ward of each pair was randomized to the intervention and one ward to the control condition by the statistical institute of our department (IMedIS). As to the nature of the intervention (implementation of RTW experts) there was no blinding.

### Statistical analysis

Baseline comparisons were undertaken using chi-squared- and *t*-tests.

The primary analysis was a comparison of days at work at 12 months after discharge between the intervention group and control group. To assess the effect of the intervention on the continuous primary outcome (days at work 12 months after discharge), a linear mixed model was fitted with ward (cluster) as a random effect term to account for non-systematic variance related to the ward and intervention group as a fixed effect using the lme4 R package. The point estimate for the intervention effect is reported together with the corresponding 95% confidence interval. A *p*-value of <0.05 was considered statistically significant. A per-protocol approach was taken to the analysis, that is, patients in intervention clusters were analyzed in this group, if they had contact with the RTW expert at least once during their inpatient stay and once after discharge. Exploratory analyses were performed to assess the effect of the intervention on the secondary outcome measures. Linear mixed models were fitted to the continuous secondary outcome measures, analogous to the primary analysis. For binary secondary outcome measures, generalized linear mixed models (logistic mixed effects models) were fitted.

### Ethics, informed consent procedure, and trial registration

The trial has been approved by the local review board (Ethikkommission der Technischen Universität München) and has been registered at Deutsches Register Klinischer Studien (DRKS00016037). All participating patients had to give written informed consent.

## Results

**Figure 1.**
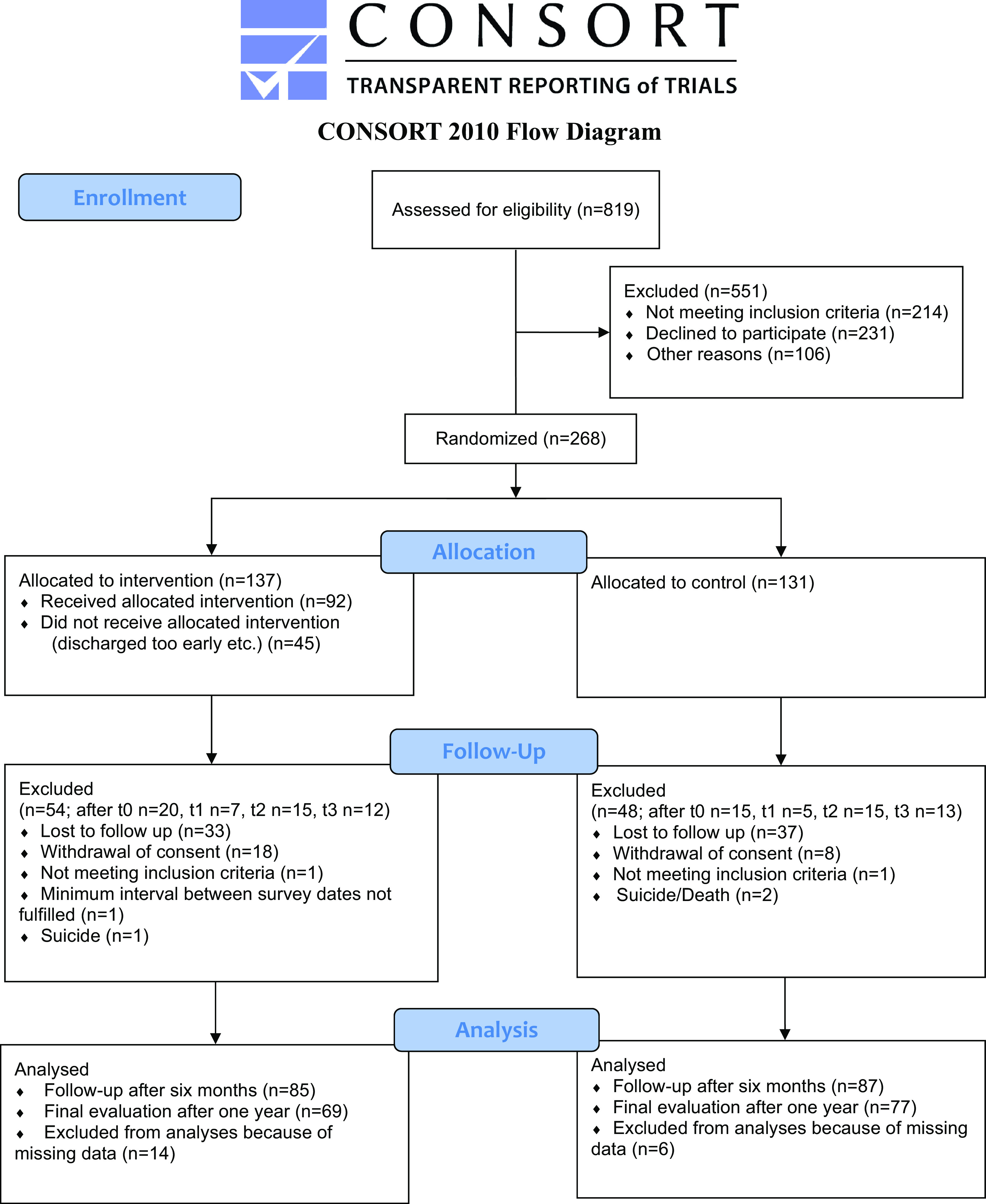
CONSORT flow diagram.

Recruitment for the RETURN study took place in 28 psychiatric wards from January 2019 until February 2020. A total of 268 patients were recruited for the trial, 137 in the intervention group and 131 in the control group. As expected, there was a considerable number of dropouts during the study period (see CONSORT diagram, Figure 1).

### Baseline characteristics

At baseline, there were no major differences between intervention group and control group regarding sociodemographic and clinical variables (see Table 1). Overall patients were around 40 years old, there were more female patients, and the most frequent diagnostic group was patients with affective disorders.

**Table 1.** Baseline characteristics of intervention group and control group: Mean (standard deviation)/frequency (%).

	Intervention *N* = 137	Control *N* = 131	Test statistic (df)	*p*
Age (years)	41.1 (10.7)	40.6 (11.5)	*t* = 0.43 (266)	0.67
Gender, female	80 (58%)	79 (60%)	*χ*^2^ = 0.04 (1)	0.85
Education			*χ*^2^ = 0.53 (1)	0.47
Up to 10 years	64 (47%)	68 (52%)		
>10 years	73 (53%)	63 (48%)		
Vocational training completed (yes)	67 (49%)	61 (47%)	*χ*^2^ = 0.07 (1)	0.79
Disability status (yes)	33 (24%)	29 (22%)	*χ*^2^ = 0.03 (1)	0.87
Duration of current employment (years)	8.9 (9.0)	9.5 (9.9)	*t* = −0.55 (265)	0.59
Duration of sick leave (days)	31.1 (60.4)	45.0 (75.8)	*t* = −1.65 (263)	0.10
Main diagnosis			*χ*^2^ = 4.33 (3)	0.23
F2	34 (25%)	27 (21%)		
F3	89 (65%)	85 (65%)		
F4	7 (5%)	10 (8%)		
F6	0 (0%)	3 (2%)		
Missing	7 (5%)	6 (5%)		
Duration of illness (years)	10.3 (10.7)	9.5 (11.1)	*t* = 0.57 (258)	0.57
Number of prev. hospitalizations	2.0 (3.0)	1.9 (2.6)	*t* = 0.10 (254)	0.92
Illness severity (CGI)	4.7 (1.0)	4.4 (0.9)	*t* = 1.68 (238)	0.09
Social functioning (GAF)	44.8 (14.5)	47.8 (13.3)	*t* = −1.70 (238)	0.09

### Clinical outcomes at discharge

At discharge, patients in both groups were comparable in terms of length of inpatient stay and most clinical outcomes. Only for the Health of the Nation Outcome Scales (HONOS) intervention patients were judged to be healthier/less socially impaired (Table 2).

**Table 2.** Clinical outcomes at discharge from hospital.

	Intervention	Control	*b*	CI (95% Wald)	*p*
Duration of inpatient treatment *n* = 200	39.2 (38.8)	49.0 (50.6)	−5.77	[−20.78;9.23]	0.452
Illness severity (CGI) *n* = 186	3.1 (1.1)	3.3 (0.9)	−0.20	[−0.61;0.21]	0.356
Social functioning (GAF) *n* = 195	62.8 (12.0)	62.0 (10.5)	0.53	[−4.43;5.50]	0.835
HONOS (behavior, impairment, symptoms, and social functioning) *n* = 159	8.7 (5.0)	11.1 (5.0)	−2.48	[−4.12;-0.83]	0.011
Mini-ICF social functioning scale *n* = 103	1.2 (1.0)	1.4 (1.0)	−0.08	[−0.57;0.42]	0.759

Abbreviations: CGI, clinical global impression; GAF, global assessment of functioning; HONOS, Health of the Nation Outcome Scales (lower values mean better health/less social impairment); Mini-ICF, mini-international classification of functioning.

### Implementation rate of support resources and patients’ readiness to return to work

Regarding standardized support resources (day clinic, RTW discussion with supervisor, stepwise reintegration, structured RTW process from the employers’ side) there was no higher uptake in the intervention group compared to the control group. Likewise, patients in the intervention group did not feel more prepared for their return to work (Table 3).

**Table 3.** Patients readiness to return to work and implementation rate of support resources.

	Intervention group	Control group	*b*	CI (95% Wald)	*p*
Fears regarding return to work (1 = strong, 5 = not at all) *n* = 173	3.1 (1.1)	3.2 (1.3)	−0.05	[−0.49;0.39]	0.82
Wish to RTW (1 = strong, 5 = not at all) *n* = 173	2.3 (1.2)	2.3 (1.2)	−0.03	[−0.44;0.39]	0.91
Prepared for RTW (1 = very good, 5 = not at all) *n* = 167	2.7 (1.0)	2.9 (1.1)	−0.27	[−0.66;0.12]	0.19
Day clinic after index hospitalization *n* = 195	22/89, 25%	30/106, 28%	−0.21	[−0.90;0.49]	0.56
Consultation with job supervisor *n* = 137	35/65, 54%	38/72, 53%	0.04	[−0.63;0.71]	0.90
Workplace health management (“BEM”)^a^ *n* = 140	30/65, 46%	26/75, 35%	0.48	[−0.20;1.16]	0.17
Part time work during sick leave (“Hamburger Modell”)^b^ *n* = 139	34/65, 52%	32/74, 43%	0.37	[−0.35;1.09]	0.32

a BEM (Betriebliches Eingliederungsmanagement): In Germany, all employers are legally required to offer BEM to all employees with longer sickness absences. The main aim of BEM is to detect causes of sickness absences and to offer specific support to employees.

b Hamburger Modell: This model of stepwise integration into work after sickness absence can be offered to all patients insured by the statutory health insurances in Germany. A stepwise integration plan is developed by patients and their physicians and has to be approved by the employer and the insurance company. Usually, patients start with a few hours per day and increase their working hours over several weeks. During stepwise integration patients are not paid by the employer but receive sickness allowances.

### Intervention effects after 6 and 12 months after discharge

Patients in the intervention group had more often returned to their workplace at 6 months (86% vs. 63%, *p* = 0.004) and at 12 months (91% vs. 81%, *p* = 0.105). Likewise, intervention patients had more days at work at 6 months (84.9 vs. 61.5, *p* = 0.014) and at 12 months (182.2 vs. 159.8, *p* = 0.143). These group differences were statistically significant at 6 months. However, for the main outcome (days at work at 12 months) these differences were no longer statistically significant. Patients in the intervention group returned to their workplace earlier than patients in the control group (*p* = 0.040). There were no significant group differences regarding quality of life or relapses (Table 4).

**Table 4.** Intervention effects 6 and 12 months after discharge.

	Intervention group	Control group	*b*	CI (95% Wald)	*p*
6 months after hospital discharge					
Patients back at work *n* = 142	57/66, 86%	48/76, 63%	1.31	[0.42; 2.21]	0.004
Days at work *n* = 154	84.9 (51.8)	61.5 (55.7)	24.01	[6.17; 41.86]	0.016
Sick leave days *n* = 144	53.2 (49.0)	72.3 (54.1)	−20.26	[−38.91; −1.61]	0.043
Quality of life (t3_pat_euroqol) *n* = 139	7.0 (2.1)	6.5 (2.3)	0.61	[−0.24; 1.45]	0.173
12 months after hospital discharge					
Patients back at work *n* = 144	59/65, 91%	64/79, 81%	0.83	[−0.18; 1.85]	0.105
Days from discharge to RTW *n* = 148	39.8 (64.1)	67.9 (92.0)	−28.31	[−55.09; −1.53]	0.040
Days at work (main outcome) *n* = 134	182.2 (85.1)	159.8 (89.6)	22.47	[−7.43; 52.38]	0.143
Sick leave days *n* = 119	78.8 (77.7)	87.9 (73.2)	−9.04	[−36.17; 18.09]	0.515
Relapse 12 months *n* = 135	38/63, 60%	52/72, 72%	−0.54	(−1.34;0.26]	0.19

## Discussion

The results of the RETURN-study show that a case-management intervention for psychiatric inpatients can improve the return to existing workplaces after discharge. Intervention group patients returned to work earlier, resulting in significant superiority over TAU in terms of the percentage of patients who went back to work, days at work, and sick leave days at 6 months follow-up. For the main outcome parameter (days at work at 12 months), however, the group difference was not significant.

### Limitations

Our study has focused on a subgroup of psychiatric inpatients, that is, those still having workplaces in the competitive labor market. Therefore, our results are not transferable to patients aiming at reentering the labor market. Here, supported employment (IPS) is an established approach [18]. In addition, the follow-up of our study fell in lockdown periods of the COVID-pandemic. Due to workplace restrictions, many patients returning to work during this time were sent to home–office which may have influenced results, most likely similarly in the intervention group and control group. Finally, most patients in our trial were suffering from severe mental illnesses. Results are therefore only partially comparable to existing studies on RTW that mainly focused on patients with common mental diseases [2]. Therefore, in our sample, the role of the “endogenic” disease might be more prominent than work-related factors such as “burnout”-symptoms [19].

### Interpretation of results

Our intervention led to patients returning earlier to their workplaces and going to work more frequently compared to control group patients at 6 months follow-up. The patients in the control group returned later to their workplace. Thus, they needed 12 months to reach the share of returned patients that the intervention group reached at 6 months. The numerical difference in days at work persisted within 12 months, however, this difference (main outcome) proved not to be statistically significant.

We believe that a number of factors might be responsible for this (negative) finding for the main outcome after 12 months. First, this might be due to a “ceiling effect” as 86% of the patients in the intervention group were already back at work after 6 months. Possibly, we had underestimated the wake of a labor market in the Munich area, which has been increasingly characterized with skills shortage—even during the Corona pandemic (which did not lead to relevant increase in unemployment rates). Second, statistical effects (large confidence intervals, lower number of observed cases at 12 months compared to 6 months) might be responsible for the negative finding.

Third, there is evidence that the recovery rate increases, when health insurance benefits end [20]. With possible sick days before the baseline survey, this might have been the case for some study participants in the control group.

Regarding potential mediators of the effects observed, we could not find the expected relationships, for example, a higher uptake of structured RTW measures or a better feeling of preparedness in the intervention group. Thus, on the one hand, it might not be the mere uptake of existing measures but rather their (case)managed implementation within a context that sees RTW as a desirable process. On the other hand, intervention patients might have suffered from an increase of anxiety when confronted with RTW [21] making them feel less prepared.

Despite the non-superiority of our intervention regarding the main outcome, our study still yielded stronger/comparable effects compared to other studies focusing on common mental disorders. Thus, for adjustment disorders, CBT alone was found not to reduce time to RTW [22], while for mood disorders, combinations of work-directed and clinical interventions were found to reduce the number of sickness absence days [23].

### Implications and conclusion

If we follow the claim that “work is a critical mental health intervention” [8], the RETURN-study has enriched the knowledge on such interventions, especially with the focus on hospitalized patients still having work contracts. For this group, it has been shown that a case management intervention has the potential to optimize some aspects of the RTW process, albeit not the main endpoint of the study, days at work after 12 months of discharge. In addition, we offered a concise intervention that can—with minimum additional training—easily be offered by social workers in psychiatric hospitals and is therefore implementable in the daily routine. The only difficulty might be securing continuity of care after discharge.

To conclude, the intervention studied has shown the potential of case-management interventions when addressing RTW. Further analyses, especially the qualitative ones, may help to better understand limitations and potential areas for improvement.

## Data Availability

The data that support the findings of this study are available from the first author upon reasonable request.
